# Dynamic changes of innate lymphoid cells in acute ST-segment elevation myocardial infarction and its association with clinical outcomes

**DOI:** 10.1038/s41598-020-61903-5

**Published:** 2020-03-20

**Authors:** Jing Li, Jing Wu, Mingyou Zhang, Yang Zheng

**Affiliations:** 1 Department of Cardiovascular disease, The First Hospital, Jilin University, Changchun, China; 2 Translational Medicine, The First Hospital, Jilin University, Changchun, China

**Keywords:** Chronic inflammation, Innate lymphoid cells

## Abstract

An increasing body of evidence has implicated the innate immune system in the causation of acute ST-segment elevation myocardial infarction (STEMI). Innate lymphoid cells (ILCs) are newly identified members of the lymphoid lineage that are important effectors of innate immunity. The role of ILCs in STEMI has not been explored. We characterized the ILCs present in peripheral blood of 176 STEMI patients and 52 controls. Patients were followed up for up to 23 months. Flow cytometry showed that the proportion of total ILCs and ILC1s were significantly increased compared with controls; contrary to ILC1s, the proportion of ILC2s among total ILCs decreased significantly during the acute phase of STEMI. ILC1s percentage was an independent predictor of major adverse cardiovascular events (MACE). On multivariate Cox regression, the 3^rd^ tertile of ILC1s was associated with a higher MACE rate compared with the 1^st^ tertile (hazard ratio: 2.26; 95% confidence interval 1.56–3.27; P = 0.014). RNA-sequencing (RNA-Seq) revealed increased expressions of interferon-γ, tumor necrosis factor-α, vascular cell adhesion molecule 1 (VCAM1), and matrix metallopeptidase 9. Moreover, as active factors secreted by ILC1s, levels of interleukin (IL)−12 and IL-18 were significantly increased in STEMI patients. Increased ILC1s in patients with STEMI was associated with poor outcomes. Our findings suggest that ILC1s may play an important role in STEMI.

## Introduction

Acute ST-segment elevation myocardial infarction (STEMI) continues to be the leading cause of death worldwide, even in the era of reperfusion therapy. Most STEMI events are attributable to plaque rupture, plaque erosion, or calcified nodule^1,2^, which are morphologically different atherosclerotic lesions. STEMI triggers rapid accumulation of millions of innate immune cells, such as monocytes, macrophages, and neutrophils. These cells are key mediators of native inflammatory response and play a critical role in atherosclerosis inflammation as well as myocardial remodeling following infarction.

Innate lymphoid cells (ILCs) are newly identified members of the lymphoid lineage that were shown to be important effectors of innate immunity^3^. ILCs are composed of “cytotoxic” ILCs (natural killer cells, NKs) and “helper” ILCs^4^. “Helper” ILCs are characterized by expression of CD127. Based on the expressions of cytokines and transcription factors, “Helper” ILCs are classified into type 1 ILCs (ILC1s), type 2 ILCs (ILC2s), and type 3 ILCs (ILC3s)^3^. ILC1s express high level of T-bet but lack expression of CD117^5^; upon stimulation by cytokines interleukin (IL)-12 and IL-18, these produce T helper (Th) 1-associated cytokine interferon (IFN)-γ^3^. ILC2s exhibit positive expression of GATA3 and upon stimulation by thymic stromal lymphopoietin (TSLP), IL-25 and IL-33 produce Th2-associated cytokines^6^, The development of ILC2s requires the presence of IL-7^7^; ILC3s express retinoid-related orphan receptor γt (RORγt) and produce IL-17 and/or IL-22^8^. ILCs are increasingly recognized as novel regulators of both acute and chronic inflammation.

The role of ILCs in the process of atherosclerosis in mouse model has been proposed. Mouse that lack T-bet, IFN-γ^9^, and IL-12^10^ were shown to exhibit reduced plaque burden, and adoptive transfer of ILC1s was shown to accelerate atherosclerosis in a mouse model^11–13^. High-fat feeding of mouse can alter the number of ILC2s and their production of cytokines. ILC2s selective genetic ablation in *Ldlr*^−/−^ mice was shown to accelerate atherosclerosis development^14^. However, no study has directly investigated the role of ILCs in human atherosclerosis. In addition, the role of ILCs in the setting of STEMI has not been explored. The main aim of this study was to investigate the various subpopulations of ILCs in STEMI patients and to explore their clinical implications and possible functional change in the setting of STEMI.

## Materials and methods

Study population. From September 2016 to May 2017, we consecutively enrolled 176 patients with de novo STEMI and 52 controls admitted to the 1st Hospital of Jilin University Heart Center. This work and samples were approved by the Institutional Review Board of 1st Hospital of Jilin University and the Research Ethical Committee of the 1st Hospital of Jilin University (authorization no. 2016–118). All experimental protocols were approved by the 1st Hospital of Jilin University. All methods were performed in accordance with the protocols. All 228 participants provided written informed consent for participation in this study. The diagnosis and treatment of STEMI were carried out according to 2009 American College of Cardiology (ACC)/American Heart Association (AHA) acute STEMI diagnostic criteria. The inclusion criteria were as follows: (1) age >18 years; (2) continuous chest pain lasting for >30 min and less than 12 hours; (3) observation of ST-segment elevation of more than 2 mm in two adjacent leads by electrocardiography (ECG); (4) increased cardiac troponin levels; (5) patients underwent emergency percutaneous coronary intervention (PCI) therapy. Patients with infectious and inflammatory disorders; active cancer, severe renal failure, significant valvular heart disease, previous myocardial infarction within 6 months, or those who presented with cardiac shock were excluded. Fifty-two matched volunteers (matched for age, sex, and cardiovascular risk factors) with no significant coronary artery stenosis, as assessed by angiography or CTA, were selected as controls. Coronary angiogram were analyzed using QAngio XA version 7.2 (Medis Medical imaging system, Leiden, the Netherlands) using conventional methods^15^. The first blood samples were obtained after the initial diagnosis and prior to medical therapy. All STEMI patients received standard pharmacological therapy in adherence to prevailing guidelines^16^, including dual antiplatelet therapy, anticoagulants, statins, β-blockers, and angiotensin-converting enzyme (ACE) inhibitors. Patients were followed-up for up to 23 months after STEMI.

### Isolation of peripheral blood mononuclear cells (PBMCs)

Peripheral blood samples (8 mL) was collected from controls (n = 52) and STEMI patients (n = 176) in ethylenediaminetetraacetic acid (EDTA) tubes prior to primary percutaneous coronary intervention procedure. All samples for the assessment of cells were processed within 12 hours of collection. PBMCs were isolated using Ficoll–Hypaque density gradient centrifugation for 30 minutes at room temperature. Then the PBMCs layer was transferred to a new tube and washed twice with phosphate-buffered saline (PBS). Finally, PBMCs were suspended in complete RPMI for subsequent experiments.

### Isolation of ILC1s from PBMCs

ILC1s were sorted from PBMCs using a BD FACSAria. ILC1s were identified as Lin cocktail^−^(CD3, CD14, CD19, and CD20), CD34^−^, CD94^−^, TCRα/β^−^, TCRγ/δ^−^, CD1a^−^, CD45^+^, CD127^+^, CRTH2^−^, and CD117^−^ cells^17,18^. FITC anti-Lin (BD Bioscience, USA), FITC anti-CD94, CD34, CD1a, TCRα/β, TCRγ/δ, APC-H7 anti-CD45, Percp-cy5.5 anti-CD127, PE-Cy7 anti-CRTH2, PE anti-CD117 (Biolegend, USA). Purity was routinely >99%. Cell viability, as determined by trypan blue staining, was >99% after isolation.

### Flow cytometric analysis

ILC1s were identified as Lin^−^CD34^−^CD94^−^TCRα/β^−^TCRγ/δ^−^CD1a^−^CD45^+^CD127^+^ CRTH2^−^CD117^−^ cells as described above. ILC2s were identified as Lin^−^CD34^−^CD94^−^TCRα/β^-^TCRγ/δ^-^CD1a^−^CD45^+^CD127^+^CRTH2^+^ cells. ILC3s were identified as Lin^−^CD34^−^CD94^−^TCRα/β^-^TCRγ/δ^-^CD1a^−^CD45^+^CD127^+^CRTH2^−^CD117^+^cells^18^. pDCs were identified as Lin^-^CD45^+^CD11c^+^CD11b^−^ cells using FITC anti-Lin, APC-H7 anti-CD45, PE anti-CD11b, and AF700 anti-CD11c (BD Bioscience, USA). mDCs were identified as Lin^−^CD45^+^CD11c^+^CD11b^+^ cells. T cells were identified as CD45^+^CD3^+^ cells using APC-H7 anti-CD45, Percp-cy5.5 anti-CD3 (BD Bioscience, USA). B cells were identified as CD45^+^CD19^+^ cells using APC-H7 anti-CD45 and APC-H7 anti-CD19 (BD Bioscience, USA). Natural killer (NK) cells were identified as CD45^+^CD56^+^ cells using APC-H7 anti-CD45, AF700 anti-CD56 (BD Bioscience, USA). Monocytes were identified as CD45^+^CD14^+^ cells using APC-H7 anti-CD45, FITC anti-CD14 (BD Bioscience, USA). For VCAM1 and MMP9 staining, PBMCs were separated from the 3^rd^ tertile group of STEMI patients and controls and stained with AF700 anti-VCAM1 and APC anti-MMP9 antibody (Biolegend, San Diego, USA). Viability was assessed by Aqua. After incubation with the respective antibodies for 30 minutes at 4 °C, cells were washed twice and subjected to flow cytometric analysis.

### RNA-sequencing (RNA-Seq) and bioinformatics analysis

Freshly sorted ILC1s were obtained from PBMCs isolated from 3 STEMI patients and 3 controls using flow cytometry. For ILC1s, a total of 100–200 cells were obtained by sorting for RNA-Seq. Cells were sorted into an Eppendorf tube containing 4 µL lysis buffer obtained from BGI Company (Wu Han, China). The Eppendorf tube was placed into liquid nitrogen directly as soon as possible after completion of sorting. The sequencing and analysis work were performed by the BGI Company. The RNA-seq data have been deposited in NCBI’s Gene Expression Omnibus (Edgar *et al*., 2002) and are accessible through GEO Series accession number GSE127853 (https://www.ncbi.nlm.nih.gov/geo/query/acc.cgi?acc=GSE127853).

### RT-qPCR validation of IFN-γ, TNF-α, VCAM1, and MMP9 expression on ILC1s

ILC1s were sorted from the PBMCs isolated from controls and STEMI patients. Total RNA was extracted, and cDNA was amplified using a REPLI-g WTA Single Cell Kit (Qiagen, Germany) according to the manufacturer’s protocol. Expressions of IFN-γ, TNF-α, VCAM1, and MMP9 mRNA were measured by quantitative real-time polymerase chain reaction using the ABI Prism StepOnePlus System (Applied Biosystems, Foster City, CA, USA). The primers were as follows:

*GAPDH* forward, 5′-TGACTTCAACAGCGACACCCA-3′; *GAPDH* reverse, 5′-CACCCTGTTGCTGTAGCCAAA-3′; *MMP9* forward, 5′- CCTGGAGACCTGAGAACCAATC-3′; *MMP9* reverse, 5′- GATTTCGACTCTCCACGCATC-3′; *TNF*-α forward, 5′-TGTAGCCCATGTTGTAGCAAACC-3′; *TNF*-α reverse, 5′-GAGGACCTGGGAGTAGATGAGGTA-3′; *IFN-*γ forward, 5′- TCTGTCAAGGGCAGTAACCTG-3′; *IFN-*γ reverse, 5′- GCCCACGACTTTGTTTTCTG-3′; *VCAM1* forward, 5′-CAGACAGGAAGTCCCTGGAA-3′; *VCAM1* reverse, 5′-TTCTTGCAGCTTTGTGGATG-3′.

The relative expressions of IFN-γ, TNF-α, VCAM1, and MMP9 were calculated using the 2^(−∆∆Ct)^ method.

### Elisa

Plasma was collected from the 3^rd^ tertile group of STEMI patients and controls. Levels of IFN-γ, TNF-α, IL-12, and IL-18 were measured using ELISA kits (Multi Sciences, Hangzhou, China) according to the manufacturer’s instructions.

### Patient follow-up

Patients were followed up during routine outpatient visits, through telephone calls, and by examination of hospital records. The median follow-up period was 502 days (interquartile range, 178–642). Major adverse cardiovascular event (MACE) was defined as the first event among the following: 1) cardiac or non-cardiac death; 2) recurrent acute coronary syndrome; 3) newly diagnosed congestive heart failure based on patient symptoms in conjunction with echocardiographic features suggestive of left ventricular dysfunction.

### Statistical analysis

Continuous variables are presented as mean  ±  standard deviation or median (interquartile range). Categorical variables are expressed as frequencies and percentages. Between-group differences with respect to categorical variables were assessed by Chi-squared test or Fisher’s exact test, as appropriate. Unpaired Student *t* test or Mann–Whitney U test was used to compare the mean values between 2 independent groups. Correlation analyses were performed using Pearson coefficient; non-normally distributed variables were log-transformed prior to analysis. Multivariable logistic regression was performed after stratification of data into tertiles to identify predictors of MACE. Variables associated with *p* values < 0.10 in univariable analysis were included in the multivariable model with backward logistic regression analysis with a threshold of *p* = 0.05. KEGG pathways (hypergeometric p-value ≤0.05) were selected as the significant enrichment pathways among all DEGs.

## Results

### Clinical characteristics

There were 176 subjects in the STEMI group and 52 subjects in the control group. The mean age of patients was 59.59 ± 11.64 years; 125 patients were male (71%), 87 patients were hypertensive (49.4%), and 33 patients were diabetic (18.7%). No significant between-group differences were observed with respect to age, gender, history of hypertension, diabetes, stroke, hyperlipidemia, heart failure, smoking, total cholesterol, high-density cholesterol (HDL), or triglyceride (TG). However, white cell count, LDL, blood glucose level and Hs-CRP in the STEMI group were higher than those in the control group (Table 1).

**Table 1:** Baseline clinical characteristics of the study population.

	STEMI (n = 176)	Control (n = 52)	P Value
Age (years)	59.59 ± 11.64	57.36 ± 9.04	0.21
Male (%)	125 (71.0%)	38 (73.1%)	0.91
BMI	25.9 ± 4.2	24.8 ± 3.3	0.09
eGFR	84.7 ± 34.2	92.3 ± 31.1	0.15
Onset to visit time <6 h (%)	114 (64.7%)	N/A	N/A
Anterior myocardial infarction	89 (50.6%)	N/A	N/A
Hypertension (%)	87 (49.4%)	24 (46.2%)	0.58
Diabetes (%)	33 (18.7%)	9 (17.3%)	0.97
Stroke (%)	10 (5.7%)	2 (3.8%)	0.87
Hyperlipidemia (%)	111 (63.1%)	29 (55.8%)	0.43
History of heart failure (%)	14 (6.3%)	2 (3.8%)	0.48
Current and former smoker (%)	57 (32.4%)	16 (30.8%)	0.75
Total cholesterol (mmol/L)	4.81 ± 1.05	4.46 ± 1.04	0.06
Triglyceride TG (mmol/L)	1.77 ± 1.09	1.57 ± 0.77	0.22
HDL (mmol/L)	1.23 ± 0.58	1.29 ± 0.44	0.49
LDL (mmol/L)	3.02 ± 0.79	2.31 ± 1.01	<0.001
Glucose (mmol/L)	7.19 ± 3.91	6.74 ± 2.74	<0.001
Hs-CRP (pg/mL)	6.8 ± 2.3	2.9 ± 1.8	<0.001
White cell count (10^9^/L)	9.68 ± 2.92	7.18 ± 1.97	<0.001
Medication at admission			
Aspirin (%)	41 (23.2%)	9 (15.8%)	0.47
Thienopyridine (%)	31 (17.6%)	0 (0%)	<0.001
ACEI/ARB (%)	44 (25.0%)	5 (8.7%)	0.02
β-receptor blocker (%)	34 (19.3%)	10 (17.5%)	1
Statin (%)	16 (9.0%)	5 (8.8%)	1

### Increase in total ILCs and ILC1s in STEMI patients within the first 12 hours of STEMI onset

**Figure 1:**
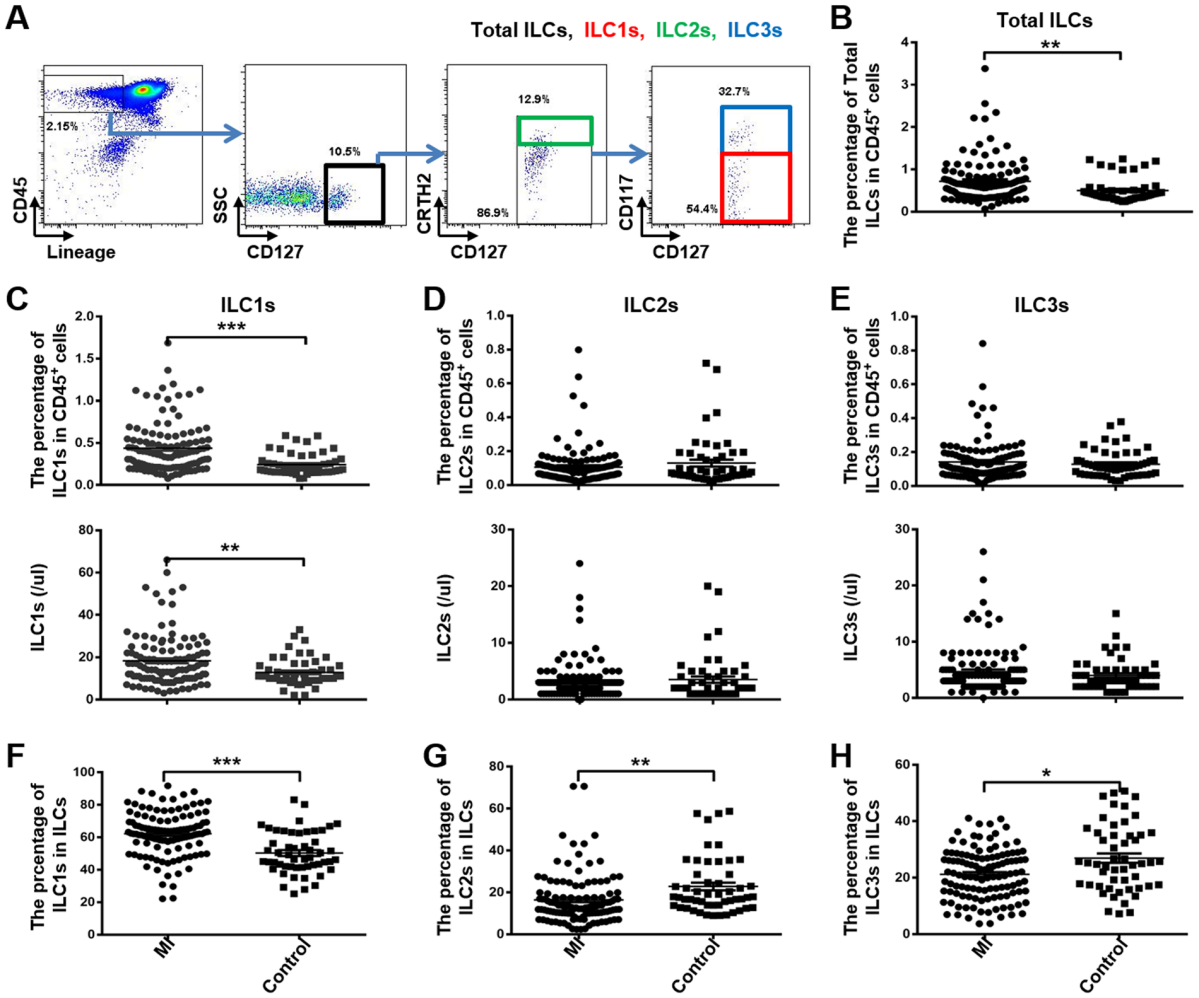
The frequency of ILC1s among PBMCs was increased in patients with STEMI. Results of FACS analysis showing the distribution of various subtypes of ILCs among the PBMCs of patients with STEMI. PBMCs were isolated from peripheral blood samples of 176 MI patients and 52 controls. (**A**) The gate of ILCs and subtypes. Total ILCs were identified as Lin^−^CD34^−^CD94^−^CD1a^−^TCRα/β^−^TCRγ/δ^−^CD45^+^CD127^+^; ILC1 were identified as Lin^−^CD34^−^CD94^−^CD1a^−^TCRα/β^−^TCRγ/δ^−^CD45^+^CD127^+^CRTH2^−^CD117^−^; ILC2 were identified as Lin^−^CD34^−^CD94^−^CD1a^−^TCRα/β^−^TCRγ/δ^−^CD45^+^CD127^+^CRTH2^+^; ILC3 were identified as Lin^−^CD34^−^CD94^−^CD1a^−^TCRα/β^−^TCRγ/δ^−^CD45^+^CD127^+^CRTH2^−^CD117^+^. (**B**) Total ILCs levels among CD45^+^ cells in STEMI patients and controls. (**C**–**H**) Percentage and number of ILC1s, ILC2s, ILC3s levels among CD45^+^ cells and total ILCs in STEMI patients and controls.

Peripheral blood samples of STEMI patients were collected within 12 hours of the onset of symptoms; blood samples of controls were obtained on the same day. Flow cytometric analysis of PBMCs showed that nearly 0.3% of CD45^+^ PBMCs exhibited an ILC phenotype (Lin^−^CD127^+^) (Fig. 1A). The percentage and number of total ILCs and ILC1s among CD45^+^ cells of STEMI patients were significantly greater than that in controls (2-fold higher) (Fig. 1B,C). No significant between-group difference was observed with respect to the percentage and number of ILC2s and ILC3s among CD45^+^ cells (Fig. 1D,E). The percentage of ILC1s among the peripheral total ILCs isolated from STEMI patients was greater than that in controls (Fig. 1F). Contrary to ILC1s, the percentage of ILC2s and ILC3s among total ILCs in STEMI patients were lower than that in controls, ILC2s are more prominent (Fig. 1G,H).

### Percentages of ILC1s subsets decreased 3 days after onset of STEMI

**Figure 2:**
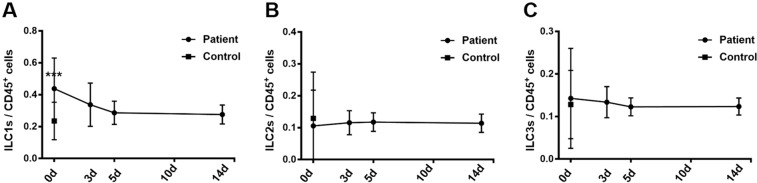
The frequency of ILCs subsets after the acute phase of STEMI. Results of FACS analysis showing the distribution of ILCs subtypes among the PBMCs of patients with STEMI. PBMCs were isolated from peripheral blood samples of STEMI patients at various time-points. (**A–C**) The percentages of ILC1s, ILC2s, and ILC3s were determined at days 0, 3, 5, and 14 after the onset of STEMI.

To monitor the change in ILCs after the STEMI event, serial blood samples were collected at days 3, 5 and 14 after the onset of STEMI. Three days after onset, the percentages of ILC1s subsets significantly decreased compared with that on day 0 (Fig. 2A); however, no changes were observed with respect to ILC2s and ILC3s (Fig. 2B,C). Compared with controls, ILC1s continued to be higher at 14 days after STEMI onset despite the decrease from day 0 (Fig. 2A).

### The increase in ILC1s was associated with the increase in monocytes and decrease in NK cells

**Figure 3:**
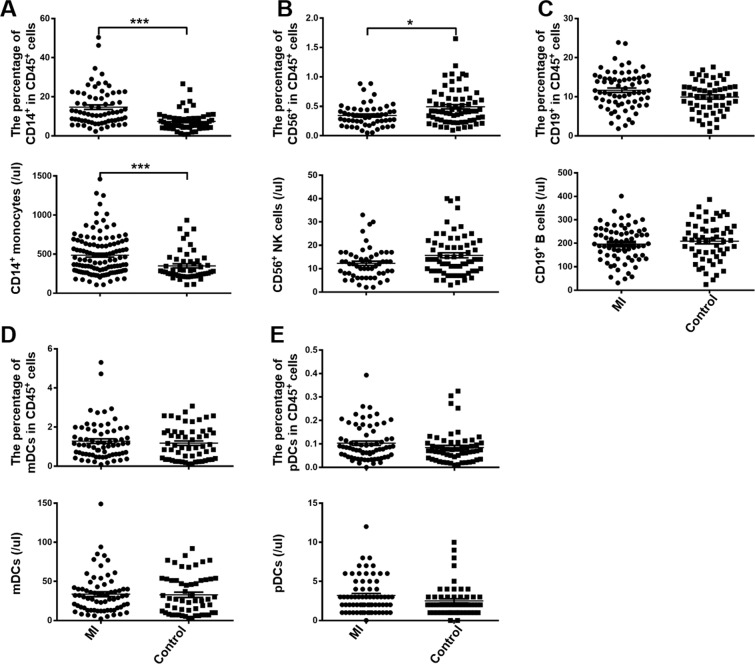
Increase in CD14^+^ monocytes and decrease in CD56^+^ NK cells among the PBMCs of patients with STEMI. Distribution of other immune cells obtained by FACS analysis. PBMCs were isolated from peripheral blood samples of 59 STEMI patients and 52 controls. (**A–E**) The percentage and number of CD14^+^ monocytes, CD56^+^ NK cells, CD19^+^ B cells, mDCs, and pDCs among CD45^+^ cells in PBMCs of STEMI patients and controls.

**Figure 4:**
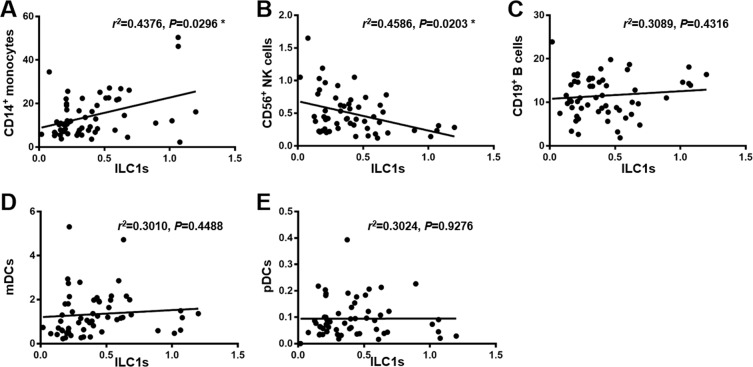
Correlation between ILC1s and CD14^+^ monocytes and CD56^+^ NK cells in STEMI patients. The correlation between ILC1s and other immune cells was analyzed. A positive correlation was observed between ILC1s and CD14^+^ monocytes (R^2^ = 0.4376, P = 0.0296). A negative correlation was observed between ILC1s and CD56^+^ NK cells (R^2^ = 0.4586, P = 0.0203). No correlation was observed between ILC1s and B cells, mDCs, and pDCs. Correlation analyses were performed using Pearson coefficient.

Monocytes, NK cells, B cells, mDCs, and pDCs were also analyzed in the current study. The percentage and number of monocytes increased significantly at day 0 (Fig. 3A). Consistent with previous reports, we observed lower levels of peripheral NK cells in STEMI patients (Fig. 3B). The B cells, mDCs, and pDCs did not show any significant change compared with control (Fig. 3C–E). On correlation analysis, ILC1s showed a positive correlation with monocytes (Fig. 4A) and a negative correlation with NK cells (Fig. 4B). The number of B cells, mDCs, and pDCs were not associated with the ILC1s (Fig. 4C–E)

### ILC1s percentage and major adverse cardiovascular events

**Figure 5:**
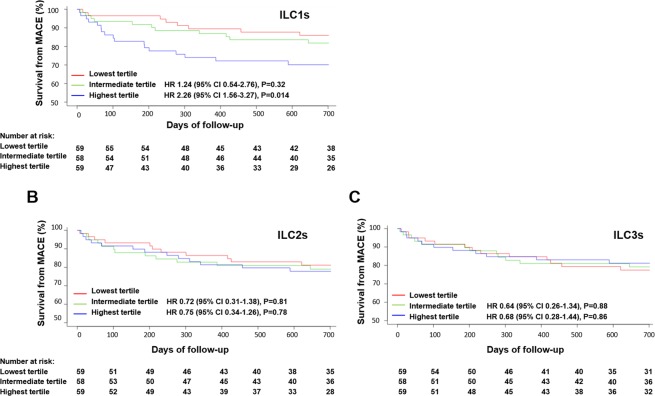
Results of Cox regression analysis showing the predictive ability of ILC subsets percentages among CD45^+^ cells for MACE after STEMI. CI, confidence interval; HR, hazard ratio; MACE, major adverse cardiovascular events; STEMI, ST-elevation myocardial infarction. Panels (**A**–**C**) MACE-free survival from MACE curves for ILC1, ILC2, and ILC3, respectively.

The linear regression was used to investigate factors associated with increased ILCs, the result showed troponin was associated with all three subsets of ILC increases, and hs-CRP was associated with ILC1s increase, but not ILC2s and ILC3s (Supplementary Table 1). The median follow-up duration was 502 days. 36 MACE occurred during follow-up. The index MACE events included 6 deaths, 16 recurrent acute coronary syndromes, and 14 cases of newly diagnosed heart failure. Based on the index ILC1s percentage among CD45^+^ cells, STEMI patients were categorized into 3 groups (Table 2). The mean percentage of ILC1s among CD45^+^ cells in the 3^rd^ tertile was 4^−^fold higher than that in the 1^st^ tertile and 2-fold higher than that in the 2^nd^ tertile. Patients in the 3^rd^ tertile had higher troponin T level. The mean Hs-CRP level in the 1^st^ tertile and 3^rd^ tertile was 4.4 ± 2.7 pg/mL and 7.6 ± 3.2 pg/mL, respectively. The hs-CRP positively correlated with percentage of ILC1s in CD45+ cells at day 0, day 3, with r = 0.34, p < 0.001; r = 0.34, p < 0.001 respectively, but not at day 5 and 14 (supplementary Table 2). On univariate Cox proportional hazard regression analysis (n = 176), the variables ILC tertile, troponin level, Hs-CRP, and LVEF were associated with a p value < 0.10; these along with diabetic variable were incorporated in the multivariate analysis. The results showed that the 3^rd^ tertile of ILC1s was also associated with a higher MACE rate compared with the 1^st^ tertile (hazard ratio, 2.26; 95% CI 1.56–3.27; P = 0.014) (Fig. 5A) (Supplementary Table 3). The percentages of both ILC2s and ILC3s were also stratified by tertiles; however, Cox regression analysis did not reveal any association of MACE risk with ILC2s and ILC3s tertiles (Fig. 5B,C).

**Table 2:** Baseline clinical characteristics of STEMI patients stratified by percentage of ILC1 in CD45^+^.

value	1st tertile N = 59	2nd tertile N = 58	3rd tertile N = 59	overall P value
ILC1% in CD45^+^ cells (%)	0.19 ± 0.05	0.36 ± 0.06	0.82 ± 0.43	<0.001
Age (years)	60.00 ± 13.08	58.32 ± 10.16	60.40 ± 11.90	0.18
Male (n)	42 (71.2%)	40 (69.0%)	43 (72.9%)	0.89
BMI (kg/m^2^)	26.2 ± 4.2	24.1 ± 4.4	25.8 ± 3.9	0.28
eGFR (mL min^−1^/1.73 m^2^)	85.6 ± 36.3	86.8 ± 29.1	84.3 ± 31.6	0.64
Onset to visit time < 6 h (n)	38 (64.4%)	36 (62.1%)	40 (67.8%)	0.44
Anterior myocardial infarction	29 (49.1%)	33 (56.9%)	26 (45.8%)	0.45
Total cholesterol (mmol/L)	4.82 ± 1.10	4.89 ± 1.05	4.75 ± 1.03	0.88
Triglyceride TG (mmol/L)	1.86 ± 1.24	1.91 ± 1.10	1.56 ± 0.93	0.26
HDL (mmol/L)	1.29 ± 0.33	1.12 ± 0.17	1.27 ± 0.29	0.34
LDL (mmol/L)	3.02 ± 0.87	2.87 ± 0.75	3.12 ± 0.77	0.35
Glucose(mmol/L)	6.59 ± 2.30	7.23 ± 2.71	7.76 ± 2.69	0.09
Gensini score	57.25 ± 27.99	64.91 ± 37.39	61.27 ± 25.69	0.37
Peak troponin T (ng/mL)	2442 ± 1897	2704 ± 2017*	3148 ± 2303*	<0.001
Creatine kinase (units/L)	982 ± 707	961 ± 788	975 ± 894	0.39
NT-pro-BNP (pg/mL)	341 ± 143	252 ± 181	316 ± 204	0.07
Hs-CRP (pg/mL)	4.4 ± 2.7	6.2 ± 2.8^§^	7.6 ± 3.2^§^	<0.001
White cell count (10^9^/L)	9.6 ± 3.1	9.4 ± 2.7	10.1 ± 2.8	0.24
LVEF (%)	51.6 ± 10.1	53.3 ± 9.2	48.7 ± 11.4	0.17
Killip class ≥ II (n)	7 (11.8%)	9 (15.5%)	7 (11.8%)	0.83
Target vessel (n)				
LAD	28 (47.5%)	34 (58.6%)	27 (45.8%)	0.32
LCX	13 (22.0%)	10 (17.2%)	12 (20.3%)	0.81
RCA	18 (30.5%)	14 (24.1%)	20 (33.9%)	0.50
Reference vessel diameter	2.94 ± 0.63	3.01 ± 0.56	2.87 ± 0.69	0.42
Lesion length	24.6 ± 17.1	21.6 ± 12.1	23.3 ± 15.3	0.64
Pre Minimal lumen diameter	0.69 ± 0.68	0.62 ± 0.63	0.71 ± 0.61	0.76
Pre diameter stenosis	79.6 ± 21.8	76.5 ± 22.3	78.0 ± 21.1	0.70
Post diameter stenosis	12.3 ± 14.7	11.9 ± 13.4	15.0 ± 15.1	0.30
Medication at discharge				
Aspirin (n)	59 (100%)	58 (100%)	59 (100%)	1
Thienopyridine (n)	54 (91.5%)	55 (94.8%)	55 (93.2%)	0.93
ACEI/ARB (n)	38 (64.4%)	35 (60.3%)	34 (57.6%)	0.74
β-receptor blocker (n)	46 (78.0%)	48 (82.8%)	45 (76.3%)	0.67
Statin (n)	59 (100%)	58 (100%)	59 (100%)	1
Outcome events (MACE)	8 (13.6%)	11 (18.9%)^¶^	17 (28.8%)^¶^	0.03
Death (n)	1 (1.7%)	2 (3.4%)	3 (5.1%)	0.59
ACS recurrence (n)	4 (6.8%)	4 (6.9%)	8 (13.6%)	0.34
Newly diagnosis HF (n)	3 (5.1%)	5 (8.6%)	6 (10.2%)	0.57

^*^P value 0.02, ^§^P value 0.01, ^¶^P value 0.07.

### Differentially expressed genes (DEGs) statistics of human ILC1s by RNA-Seq

RNA-Seq technique was used to further investigate the gene expression on ILC1s in STEMI patients; patients with normal coronary artery angiograms served as controls. More than 70 million clean reads were obtained from each sample group after elimination of low-quality reads. The Q20 score was above 99% for all and the mapping rate to reference genome of each sample varied from 76.07% to 85.81% (Supplementary Table 4). The data indicated that all RNA-Seq samples qualified for further analysis.

**Figure 6:**
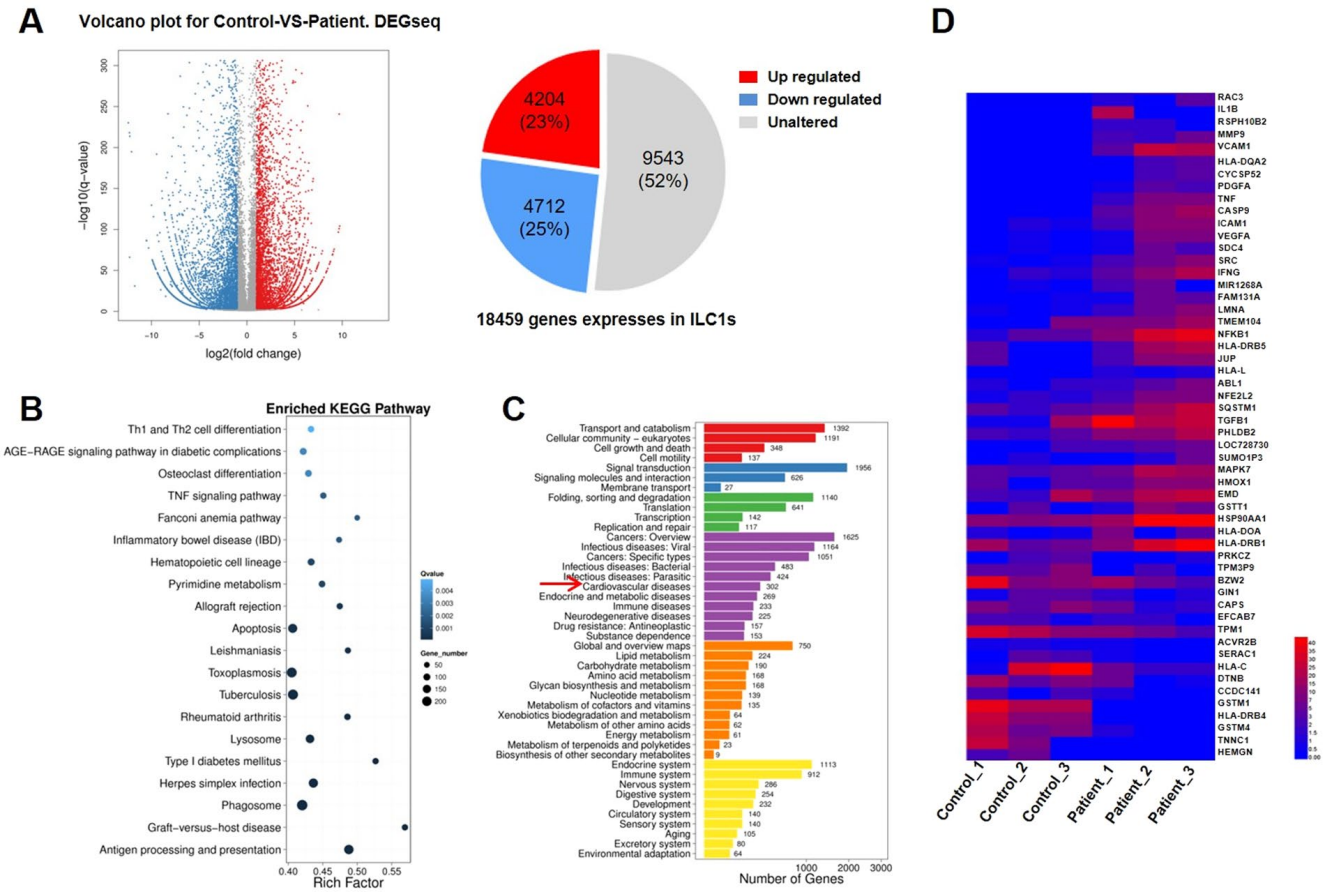
Several DEGs on ILC1s were associated with cardiovascular diseases. Based on the gene expression level, we identified the DEGs between groups. (**A**) Scatterplot of DEGs: X Y axis represents log10 transformed gene expression levels. Red color indicates the up-regulated genes; blue color represents the down-regulated genes; gray color represents the non-DEGs. (**B**) Pathway functional enrichment of DEGs. X-axis represents enrichment factor. Y-axis represents pathway name. The color indicates the q-value (high: white, low: blue); the lower the q-value the more significant is the enrichment. Point size indicates DEG number (the bigger dots refer to larger amount). Rich Factor refers to the value of enrichment factor. The larger the value, the more significant is the enrichment. (**C**) Pathway classification of DEGs. X-axis represents the number of DEGs; Y-axis represents functional classification. (**D**) RNA Seq analysis of DEGs on comparison of ILCs between STEMI patients and controls. Data from the PBMC samples of 3 STEMI patients and 3 controls are shown.

A total of 4204 DEGs were found up-regulated and 4712 DEGs down-regulated on comparison of ILC1s from STEMI patients and controls (Fig. 6A). Kyoto Encyclopedia of Genes and Genomes (KEGG) were utilized to confirm significantly enriched antigen processing and presentation pathway and apoptosis pathway (Fig. 6B). A total of 302 DEGs were related to cardiovascular diseases (Fig. 6C). After removal of BGI novel genes, 222 genes were associated with cardiovascular diseases. On further analysis, 89 genes showed ≥2 Log fold change (Log^2^ ratio) and 48 genes showed <−2 Log fold change (Log^2^ ratio) (data do not show). The number of up-regulated genes was far more than that of the down-regulated genes. The results showed that at the time of STEMI event not only ILC1s numbers changes, their corresponding gene expressions also undergo distinctive change, which indicating active involvement of ILCs in STEMI.

In addition to log values, sample FPKM-value variation was also taken into consideration. Finally, we identified 17 down-regulated and 36 up-regulated genes with certain known cardiovascular related functions after comparison of ILC1s isolated from MI patients and controls (Fig. 6D) (Supplementary Tables 5, 6).

Among the 36 up-regulated genes, multiple genes related to fluid shear stress, atherosclerosis, and platelet activation were identified. Functional analysis showed that genes related to antigen processing and presentation included *HLA-DRB1, HLA-DOA*; genes related to Th1 and Th2 cell differentiation included *HLA-DRB1, HLA-DOA, HLA-DRB5, NFKB1, IFN-ɣ*, and *RSPH10B2* (Supplementary Table 5).

The 17 down-regulated genes included genes associated with adrenergic signaling in cardiomyocytes; genes associated with cardiac muscle contraction included *TNNC1, TPM1*, and *TPM3P9*; genes associated with shear stress and atherosclerosis included *GSTM4, GSTM1, ACVR2B, EFCAB7, CAPS*, and *PRKCZ*. Functional analysis showed that gene *PRKCZ* is associated with platelet activation; *HLA-DRB4* and *HLA-C* genes are associated with antigen processing and presentation; *SERAC1* associated with MAPK signaling pathway; TGF-beta signaling pathway included *ACVR2B*; cAMP signaling pathway included *EFCAB7* and *CAPS*; cGMP-PKG signaling pathway included CAPS; Phosphatidylinositol signaling system included CAPS; mTOR signaling pathway included GIN1 (Supplementary Table 6).

**Figure 7:**
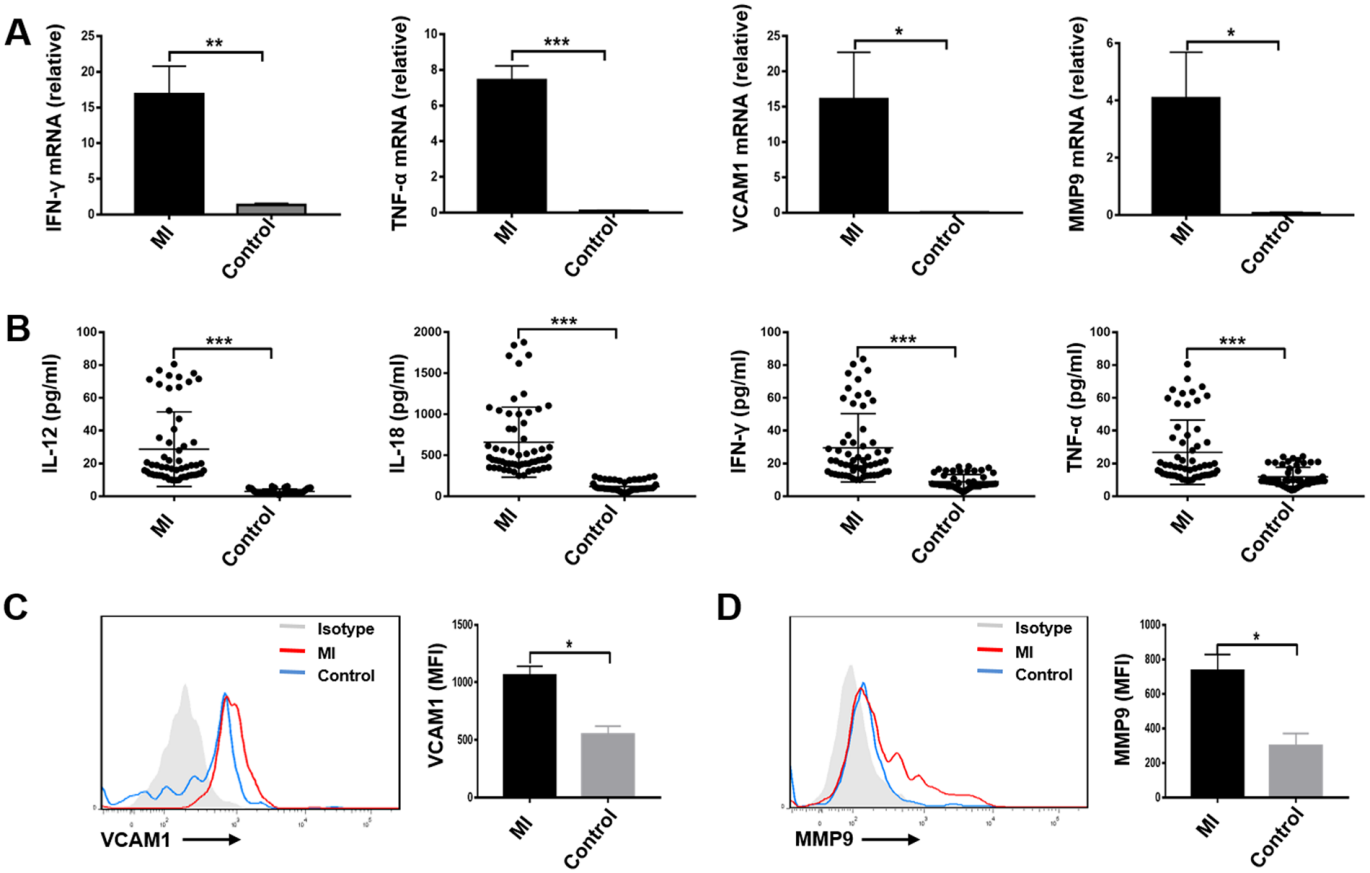
(**A**) The expressions of IFN-γ, TNF-α, VCAM1, and MMP9 on ILC1s. Real-time RT-PCR assays for IFN-γ, TNF-α, VCAM1, and MMP9 on ILC1s from 3^rd^ tertile group of STEMI patients and controls (n = 6). The expression levels were calculated using the 2^(−∆∆Ct)^ method. (**B**) The release of IFN-γ, TNF-α, IL-12, and IL-18 from STEMI patients. Plasma was collected from 3^rd^ tertile group of STEMI patients and controls and measured by Elisa. (**C**) The expressions of VCAM1 and MMP9 on ILC1s. PBMCs were separated from 3^rd^ tertile group of STEMI patients and controls and measured by flow cytometry.

Furthermore, we selected samples from 6 randomly selected patients in the 3^rd^ ILC1s tertile group to detect the expressions of IFN-γ, TNF-α, VCAM1, and MMP9 on ILC1s by RT-qPCR. Consistent with the RNA-seq, the expressions IFN-γ, TNF-α, VCAM1, and MMP9 on ILC1s were significantly higher than those in the control group (Fig. 7A).

In addition, we further detected ILC1s active factors IL-12, IL-18, and effective factors IFN-γ and TNF-α in the plasma from ILC1s 3^rd^ tertile group of STEMI patients and controls by Elisa. The results showed these cytokines were significantly increased in STEMI patients (Fig. 7B). We also detected the expressions of VCAM1 and MMP9 on ILC1s by flow cytometry. The expressions of (C) VCAM1 and (D) MMP9 on ILC1s from STEMI patients were higher than those in controls (Fig. 7C,D). These results were consistent with the results of RNA-seq.

## Discussion

To our best knowledge, this is the first study that investigated the role of ILCs in STEMI patients. We found significantly higher proportions of total ILCs and its subgroup ILC1s in STEMI patients as compared to those in controls. Among STEMI patients, high percentage of ILC1s was associated with poor clinical outcomes.

ILCs are a recently discovered addition to the expanding family of immune cells that consists of several distinct subtypes. Studies have shown that different subtypes may have divergent effects in atherosclerosis^12^. Increased accumulation of ILC1s in atherosclerotic lesions was reported in mouse model^11^. In the present study, total ILCs increased in the acute phase of STEMI. The increase in total ILCs was largely driven by the increase in ILC1s alongside a significant decrease in ILC2s. The different response of different subtypes is consistent with the hypothesis that different subtypes may have divergent effects on atherosclerosis^12^.

ILCs rapidly produce pro-inflammatory and regulatory cytokines in response to the production of cytokines or signals expressed by tissue-resident cells^19,20^. The most dramatic increase in total ILCs and ILC1s subtype was observed on day 0 of STEMI. In addition, the increase was associated with a proportionate decrease in NK cells. ILC1s along with the NK cells are referred to as group 1 ILCs^21^. Group 1 ILCs are distinguished from other ILCs based on rapid production of IFN-γ following stimulation^22,23^. In a mice model, the lack of IL-12 and IFN-γ was shown to be associated with reduced plaque burden^9^. In earlier studies, depletion of NK cells was shown to reduce atherosclerosis while NK cell transfers increased plaque formation^24,25^. Thus, it is reasonable to speculate that ILC1s may contribute to lesion development, aggravate local inflammation, and promote plaque instability that may trigger a STEMI event. Furthermore, the proportion of ILC1 among STEMI patients is associated with Peak Troponin and Hs-CRP level. Peak Troponin reflects infarction size and predicts prognosis^26,27^, Hs-CRP is a well-recognized inflammatory maker^28^. These findings resonate with the link of ILC1s with the clinical outcomes. ILCs coordinate with other innate as well as adaptive immune cells may mount dynamic response involved in heart remodeling^29^. While no sufficient data to conclude the detrimental or protective role of ILC1s in STEMI, its early increase, and association with clinical outcomes are interesting and warrant further investigation. For the future study, patient with stable angina, unstable angina, and NSTEMI should also be enrolled to further illuminate the role of ILC1s.

RNA-Seq technique is a new-generation transcriptome sequencing technique that can provide unprecedented knowledge of gene expression in specific immune cells^30^. The study of gene expression profile of ILC1s offers good prospects for further understanding the role of ILC1s in the STEMI setting. In our study, distinctive gene expression profiles were observed in STEMI patients compared with controls. In the enriched KEGG analysis, antigen processing and presentation pathway and apoptosis pathway genes were significantly enriched, but not Th1 and Th2 cell differentiation. One possible explanation of this observation could be the early involvement of ILCs, as these represent an important component of the innate immune response; unlike adaptive lymphocytes, ILCs can quickly respond to stimulation.

As expected, among the cardiovascular disease related genes, *MMP9, TNF, IFN-ɣ*, and *VCAM1* genes were the top up-regulated genes in ILC1s was earlier shown to play an important role in the occurrence of post-PCI restenosis. Higher levels of TNF, IFN-γ, and MMP-9 in the early stages were associated with the extent of LV remodeling after AMI^31,32^. Elsewhere, STEMI patients with high VCAM levels were shown to be at an increased risk of death and MI^33,34^. RNA expressions of these genes were further confirmed by RT-qPCR. The evident up-regulation of these proinflammatory cytokines in STEMI patients suggested fundamental functional changes in ILC1s. Collectively, these findings explain the observed association between high percentages of ILC1s and poor outcomes.

One of the key limitations of this study is that the assessments were based on peripheral blood; it is not known how closely these parameters reflect the process within the plaque as well as the myocardium. Lack of group of stable CAD is another limitation of this study, for the future study, it will be interesting to see the changes of ILCs in stable CAD patients. Owing to the relatively small sample size, the study is underpowered for detection of each MACE component. Further investigations of the regulatory mechanisms and the mechanism underlying its association with prognosis are also warranted.

## Conclusions

In summary, the dynamic functional changes in ILCs (especially the ILC1s subtype) at the time of STEMI onset and its observed association with outcomes indicate an important role of ILCs in STEMI. This study may better characterize the role of ILCs in STEMI and may potentially unravel novel therapeutic targets for the treatment of STEMI.

## Supplementary information

Supplementary Materials.

## Data Availability

All data arising from this study are contained within the manuscript information files.
