# Supplementary material for: Dynamic changes of innate lymphoid cells in acute ST-segment elevation myocardial infarction and its association with clinical outcomes
Source: Sci Rep. 2020 Mar 20;10:5099. doi: 10.1038/s41598-020-61903-5 (PMC7083894; doi:10.1038/s41598-020-61903-5)
Supplement: Supplementary file 1 — Supplementary Materials. [file 41598_2020_61903_MOESM1_ESM.docx]

**Dynamic changes of innate lymphoid cells in acute ST-segment elevation myocardial infarction and its association with clinical outcomes**

Jing Li^1^, Jing Wu^2^, Mingyou Zhang^1*^& Yang Zheng^1*^

^1^Department of Cardiovascular disease, The First Hospital, Jilin University, Changchun, China

^2^Translational Medicine, The First Hospital, Jilin University, Changchun, China

Correspondence should be addressed to Mingyou Zhang: zmy@jlu.edu.cn, and Yang Zheng: [Zhengyanghappy07@sina.com](mailto:Zhengyanghappy07@sina.com)

| **Table S1. The association between clinical factors and the percentage of ILCs in CD45^+^ cells**  **Variable Coefficient and CI** **P value** | | | |
| --- | --- | --- | --- |
| ILC1 | LVEF (per 10%) | 0.04 (-0.1, 0.06) | 0.11 |
|  | Troponin (per 1000ng/ml) | 0.06 (0.02, 0.12) | 0.03 |
|  | eGFR (per 10mL min-1/1.73m^2^) | 0.06 (-0.19, 0.31) | 0.64 |
|  | glucose (per 1 mg/dl) | 0.01 (-0.02, 0.03) | 0.59 |
|  | hs CRP (per 1 mg/ml) | 0.04 (0.02, 0.07) | < 0.001 |
|  | Age (per year) | 0.01 (-0.01,0.02 ) | 0.92 |
|  | Male sex (vs female) | 0.06 (-0.09, 0.20) | 0.42 |
|  | Hyperlipedemia | 0.08 (-0.06, 0.20) | 0.24 |
|  | Diabetes | 0.10 (-0.07, 0.27) | 0.23 |
|  |  |  |  |
| ILC2 | LVEF (per 10%) | 0.02 (-0.16, 0.22) | 0.43 |
|  | Troponin (per 1000ng/ml) | 0.04 (0.02, 0.10) | 0.03 |
|  | eGFR (per 10mL min-1/1.73m^2^) | 0.01 (-0.01, 0.03) | 0.89 |
|  | glucose (per 1 mg/dl) | 0 (-0.01, 0.01) | 0.85 |
|  | hs CRP (per 1 mg/ml) | 0 (-0.01, 0.01) | 0.87 |
|  | Age (per year) | 0 (0,0) | 0.66 |
|  | Male sex (vs female) | 0.03 (-0.01, 0.08) | 0.17 |
|  | Hyperlipedemia | 0.03 (-0.02, 0.07) | 0.34 |
|  | Diabetes | 0.02 (-0.04, 0.07) | 0.51 |
|  |  |  |  |
| ILC3 | LVEF (per 10%) | 0.02 (-0.1, 0.06) | 0.08 |
|  | Troponin (per 1000ng/ml) | 0.02 (0.02, 0.06) | 0.04 |
|  | eGFR (per 10mL min-1/1.73m^2^) | 0 (-0.19, 0.31) | 0.28 |
|  | glucose (per 1 mg/dl) | 0.01 (-0.02, 0.03) | 0.19 |
|  | hs CRP (per 1 mg/ml) | 0.01 (0.02, 0.07) | 0.002 |
|  | Age (per year) | 0 (0,0 ) | 0.9 |
|  | Male sex (vs female) | 0.05 (-0.08, 0.18) | 0.24 |
|  | Hyperlipedemia | 0.02 (-0.02, 0.05) | 0.39 |
|  | Diabetes | 0.02 (-0.03, 0.06) | 0.4 |

| **Table S2. Correlation between hs-CRP and percentage of ILC1 in CD45^+^ cells at different time points**  **r P value** | | |
| --- | --- | --- |
| day 0 | 0.34 | <0.001 |
| day 3 | 0.27 | 0.03 |
| day 5 | 0.17 | 0.12 |
| day 14 | 0.16 | 0.35 |

| **Table S3. Univariate and multivariate predictors for major cardiac adverse events**  **Univariate P value Multivariable P value** | | | | | | | |
| --- | --- | --- | --- | --- | --- | --- | --- |
|  |  | HR (95% CI) | |  | HR (95% CI) | |  |
| ILC1s (per 1 %) | highest tertile vs. lowest tertile | 2.53 (1.29, 3.76) | 0.003 | | 2.26 (1.56, 3.27) | 0.01 | |
|  | intermediate tertile vs. lowest tertile | 1.35 (0.48, 2.22) | 0.14 | | 1.24 (0.54, 2.76) | 0.32 | |
| ILC2s (per 1 %) | highest tertile vs. lowest tertile | 0.89 (0.47, 1.52) | 0.65 | | 0.75 (0.34, 1.26) | 0.78 | |
|  | intermediate tertile vs. lowest tertile | 1.04 (0.51, 1.48) | 0.57 | | 0.72 (0.31, 1.38) | 0.81 | |
| ILC3s (per 1 %) | highest tertile vs. lowest tertile | 0.95 (0.38, 1.32) | 0.49 | | 0.68 (0.28, 1.44) | 0.86 | |
|  | intermediate tertile vs. lowest tertile | 0.92 (0.36, 1.47) | 0.83 | | 0.64 (0.26, 1.34) | 0.88 | |
| Troponin (per 1000ng/ml) | | 2.57 (1.14, 3.91) | 0.001 | | 2.67 (1.44, 3.51) | 0.02 | |
| hs CRP (per 1 mg/ml) | | 1.59 (1.12, 2.05) | 0.04 | | 1.87 (0.95, 2.31) | 0.13 | |
| LVEF (per 10%) |  | 1.86 (1.31, 2.44) | 0.02 | | 1.68 (1.19, 2.07) | 0.04 | |

| **Table S4.  Clean reads quality metrics**  **Sample Total clean reads (M) Clean reads Q20 (%) Clean reads ration (%)** | | | |
| --- | --- | --- | --- |
| Control_1 | 67.12 | 99.03 | 85.81 |
| Control_2 | 73.68 | 99.02 | 77.56 |
| Control_3 | 71.85 | 99.03 | 80.24 |
| Patient_1 | 74.81 | 99.06 | 78.75 |
| Patient_2 | 73.73 | 99.04 | 79.50 |
| Patient_3 | 73.99 | 99.09 | 76.07 |

| **Table S5. 36 up regulated DEGs related to cardiovascular diseases in STEMI patients**  **Gene symbol Ensembl ID Gene name Log2 fold change P value** | | | | |
| --- | --- | --- | --- | --- |
| RAC3 | 5881 | Rac family small GTPase 3 | +8.19 | 9.11E-25 |
| VCAM1 | 7412 | Vascular cell adhesion molecule 1 | +7.99 | 3.37E-22 |
| RSPH10B2 | 728194 | Radial spoke head 10 homolog B2 | +7.66 | 1.96E-18 |
| MMP9 | 4318 | Matrix metallopeptidase 9 | +7.65 | 1.55E-68 |
| HLA-DQA2 | 3118 | Major Histocompatibility Complex, Class II, DQ Alpha 2 | +7.30 | 7.77E-32 |
| CYCSP52 | 360155 | Cytochrome c, somatic pseudogene 52 | +6.90 | 4.13E-12 |
| PDGFA | 5154 | Platelet derived growth factor subunit A | +6.76 | 1.87E-97 |
| TNF | 7124 | Tumor necrosis factor | +6.30 | 0.000000 |
| CASP9 | 842 | Caspase 9 | +6.19 | 0.000000 |
| ICAM1 | 3383 | Intercellular Adhesion Molecule 1 | +4.70 | 0.000000 |
| VEGFA | 7422 | Vascular endothelial growth factor A | +4.57 | 2.74E-226 |
| SDC4 | 6385 | Syndecan 4 | +4.46 | 1.50E-121 |
| SRC | 6714 | SRC | +4.28 | 7.88E-303 |
| IFNG | 3458 | Interferon gamma | +4.20 | 0.000000 |
| MIR1268A | 100302233 | MicroRNA 1268a | +4.18 | 1.14E-43 |
| FAM131A | 131408 | Family With Sequence Similarity 131 Member A | +4.17 | 6.54E-82 |
| LMNA | 4000 | Lamin A/C | +4.03 | 0.000000 |
| TMEM104 | 54868 | Transmembrane protein 104 | +3.59 | 0.000000 |
| NFKB1 | 4790 | Nuclear factor-kappa B1 | +3.39 | 0.000000 |
| HLA-DRB5 | 3127 | Major Histocompatibility Complex Class II, DRβ1 and 5 | +3.35 | 2.49E-203 |
| JUP | 3728 | Junction plakoglobin | +3.18 | 0.000000 |
| HLA-L | 3139 | Major Histocompatibility Complex, Class I, L | +2.85 | 1.46E-31 |
| ABL1 | 25 | ABL proto-oncogene 1 | +2.81 | 8.07E-268 |
| NFE2L2 | 4780 | Nuclear Factor, Erythroid 2 Like 2 | +2.76 | 0.000000 |
| SQSTM1 | 8878 | Sequestosome 1 | +2.71 | 0.000000 |
| TGFB1 | 7040 | Transforming growth factor beta 1 | +2.64 | 4.97E-05 |
| PHLDB2 | 90102 | Pleckstrin Homology Like Domain Family B Member 2 | +2.62 | 0.000000 |
| LOC728730 | 728730 | Uncharacterized LOC728730 | +2.58 | 1.18E-24 |
| SUMO1P3 | 474338 | SUMO1 pseudogene 3 | +2.57 | 3.30E-26 |
| MAPK7 | 5598 | Mitogen-activated protein kinase 7 | +2.54 | 0.000000 |
| HMOX1 | 3162 | Heme oxygenase 1 | +2.47 | 0.000000 |
| EMD | 2010 | Emerin | +2.28 | 4.34E-218 |
| GSTT1 | 2952 | Glutathione S-Transferase Theta 1 | +2.27 | 1.21E-51 |
| HSP90AA1 | 3320 | Heat shock protein 90 alpha family class A member 1 | +2.14 | 0.000000 |
| HLA-DOA | 3111 | Major histocompatibility complex, class II, DO alpha | +2.11 | 3.24E-46 |
| HLA-DRB1 | 3123 | Major Histocompatibility Complex, Class II, DR Beta 1 | +2.01 | 0.000000 |

| **Table S6. 17 down regulated DEGs related to cardiovascular diseases in STEMI patients**  **Gene symbol Ensembl ID Gene name Log2 fold change P value** | | | | |
| --- | --- | --- | --- | --- |
| HEMGN | 55363 | Hemopoietic gene protein | -8.64 | 7.85E-58 |
| TNNC1 | 7134 | Troponin C1 | -6.64 | 3.83E-68 |
| GSTM4 | 2948 | Glutathione S-Transferase Mu 4 | -6.08 | 8.76E-183 |
| HLA-DRB4 | 3126 | Major histocompatibility complex, class II, DR beta 4 | -5.27 | 0.000000 |
| GSTM1 | 2944 | Glutathione S-transferase Mu 1 | -4.78 | 9.11E-280 |
| CCDC141 | 285025 | Coiled-Coil Domain Containing 141 | -4.43 | 6.04E-98 |
| DTNB | 1838 | Dystrobrevin Beta | -4.39 | 1.63E-121 |
| HLA-C | 3107 | Major Histocompatibility Complex, Class I, C | -4.00 | 0.000000 |
| SERAC1 | 84947 | Serine Active Site Containing 1 | -3.37 | 2.51E-70 |
| ACVR2B | 93 | Activin A receptor type 2B | -3.30 | 4.83E-89 |
| TPM1 | 7168 | Tropomyosin 1 | -2.67 | 0.000000 |
| EFCAB7 | 84455 | EF-Hand Calcium Binding Domain 7 | -2.49 | 1.88E-30 |
| CAPS | 828 | Calcyphosine | -2.42 | 3.62E-99 |
| GIN1 | 54826 | Gypsy retrotransposon integrase-like protein 1 | -2.16 | 1.75E-47 |
| BZW2 | 28969 | Basic Leucine Zipper and W2 Domains 2 | -2.15 | 5.85E-195 |
| TPM3P9 | 147804 | Tropomyosin 3 pseudogene 9 | -2.10 | 4.85E-89 |
| PRKCZ | 5590 | Protein kinase C zeta | -2.00 | 2.25E-24 |
